# Cough Medicines

**Published:** 1874-10

**Authors:** 


### COUGH MEDICINES.

For a generation we have advocated the avoidance of all cough medicines in colds and consumption, because the tickling in the throat which excites coughing is the result of something being there or in the lungs, which ought to be removed, and coughing is nature's method of effecting this removal. Instead then of repressing cough, it would be better to promote it, or do something to loosen or dilute the phlegm, so that it may be coughed up more readily. The less a consumptive coughs, the more is he troubled with shortness of breath, because that which would be otherwise coughed up remains in the lungs, incessantly accumulating, thus filling them up and preventing the air getting in. In all cases of consumption, the cough begins to abate a day or two before the end, because there is not strength to cough; the yellow matter fills up the lungs, and death closes the scene.

In a lifetime we have just first seen in print a confirmation of our views in the London *Lancet*, the standard medical periodical among English speaking nations, of June 13, 1874. "Anodynes, narcotics, cough mixtures and lozenges are practically of no good, and but too often increase the debility and hasten the fatal end." And yet millions of money are spent every year in the purchase of cough medicines and soothing syrups, with special editorial advocacies in all forms and phases, written by accommodating editors, political and religious, thus filching from multitudes of families the last hard earned dollar for a bottle of "cough medicine," when that dollar was sorely needed for bread; but willingly given by affectionate hearts because it eases the cough, but only the sooner to kill, in every case, without exception.

The best method of easing cough is to resist it with all the force of will possible, until the accumulation of phlegm becomes greater, then there is something to cough against, and it comes up very much easier, and with half the coughing. A great deal of hacking, and hemming, and coughing in invalids is nervous, purely nervous, or from the force of habit, as is shown by the frequency when thinking about it, and the comparative rarity, when the person is so much engaged that there is no time to think about it, and the attention is compelled in another direction.

